# The efficacy of non-invasive brain stimulation in the treatment of children and adolescents with Anorexia Nervosa: study protocol of a randomized, double blind, placebo-controlled trial

**DOI:** 10.1186/s40337-023-00852-6

**Published:** 2023-08-02

**Authors:** Luciana Ursumando, Viviana Ponzo, Alessio Maria Monteleone, Deny Menghini, Elisa Fucà, Giulia Lazzaro, Romina Esposito, Silvia Picazio, Giacomo Koch, Valeria Zanna, Stefano Vicari, Floriana Costanzo

**Affiliations:** 1 Child and Adolescent Neuropsychiatry Unit, Department of Neuroscience, Bambino Gesù Children’s Hospital, IRCCS, Piazza Sant’Onofrio 4, 00165 Rome, Italy; 2 Neurosurgery Unit, Department of Neuroscience, Bambino Gesù Children’s Hospital, IRCCS, Rome, Italy; 3 Department of Psychiatry, University of Campania “Luigi Vanvitelli”, Naples, Italy; 4 Experimental Neuropsychophysiology Lab, IRCCS S. Lucia Foundation, Rome, Italy; 5 Department of Psychology, University “Sapienza” of Rome, Rome, Italy; 6 Section of Human Phisiology, University of Ferrara, Ferrara, Italy; 7 Department of Life Science and Public Health, Catholic University of the Sacred Heart, 00168 Rome, Italy

**Keywords:** Eating disorders, AN, Neuromodulation, tDCS, TMS, EEG, Cortisol

## Abstract

**Background:**

Current psychological and pharmacological treatments for Anorexia Nervosa (AN) provide only moderate effective support, and there is an urgent need for research to improve therapies, especially in developing age. Non-invasive brain stimulation has suggested to have the potential to reducing AN symptomatology, via targeting brain alterations, such as hyperactivity of right prefrontal cortex (PFC). We suppose that transcranial direct current stimulation (tDCS) to the PFC may be effective in children and adolescents with AN.

**Methods:**

We will conduct a randomized, double blind, add-on, placebo-controlled trial to investigate the efficacy of tDCS treatment on clinical improvement. We will also investigate brain mechanisms and biomarkers changes acting in AN after tDCS treatment. Eighty children or adolescent with AN (age range 10–18 years) will undergo treatment-as-usual including psychiatric, nutritional and psychological support, plus tDCS treatment (active or sham) to PFC (F3 anode/F4 cathode), for six weeks, delivered three times a week. Psychological, neurophysiological and physiological measures will be collected at baseline and at the end of treatment. Participants will be followed-up one, three, six months and one year after the end of treatment. Psychological measures will include parent- and self-report questionnaires on AN symptomatology and other psychopathological symptoms. Neurophysiological measures will include transcranial magnetic stimulation (TMS) with electroencephalography and paired pulse TMS and repetitive TMS to investigate changes in PFC connectivity, reactivity and plasticity after treatment. Physiological measures will include changes in the functioning of the endogenous stress response system, body mass index (BMI) and nutritional state.

**Discussion:**

We expect that tDCS treatment to improve clinical outcome by reducing the symptoms of AN assessed as changes in Eating Disorder Risk composite score of the Eating Disorder Inventory-3. We also expect that at baseline there will be differences between the right and left hemisphere in some electrophysiological measures and that such differences will be reduced after tDCS treatment. Finally, we expect a reduction of endogenous stress response and an improvement in BMI and nutritional status after tDCS treatment. This project would provide scientific foundation for new treatment perspectives in AN in developmental age, as well as insight into brain mechanisms acting in AN and its recovery.

*Trial registration* The study was registered at ClinicalTrials.gov (ID: NCT05674266) and ethical approval for the study was granted by the local research ethics committee (process number 763_OPBG_2014).

## Introduction

### Background and rationale

Anorexia Nervosa (AN) is an Eating Disorder (ED) that involves significant biological, psychological, and social complications, typically associated with other severe physical and psychological comorbidities. Mortality and disability rates are high, and the incidence among children and adolescents is increasing [1, 2].

Treatment outcomes for AN remain modest with a high risk of relapse [3–5] and there is no specific Food and Drug Administration pharmacological indication for AN [6]. Although family-based or cognitive-behavioral therapy is widely considered the treatment of first choice [7], no single recommended psychological intervention has demonstrated clear superiority in treating adults or adolescents with AN [8, 9], so the effectiveness of multidisciplinary programs has been suggested [10]. Nevertheless, the development of new and more effective treatments for AN is highly claimed, especially in developmental age.

The emergence of neurobiological models of AN has opened the opportunity for brain-directed treatment approaches. Literature has reported brain abnormalities in AN, particulary in the dorsolateral prefrontal cortex (DLPFC) [11, 12], cingulate cortex and left middle occipital gyrus [13]. Studies of functional magnetic resonance imaging show wide variability. In the context of food cue, a hyperactivity in reward-related regions [14, 15] and a hyperactivity of right DLPFC [16] following exposure to palatable food cues have been reported, suggesting elevated top-down inhibition of reward processing [17–19]. Indeed, DLPFC plays a key role in cognitive control [20, 21], in self-control in a dietary context [22, 23] and in regulating the valence of emotional experiences [24].

Based on these studies, non-invasive brain stimulation techniques (NIBS), which may directly modulate neural circuits by enhancing or reducing the excitability of key brain regions, have been proposed as therapeutic option, for example to restore the balance between right and left DLPFC activity (for a recent review see [25, 26]). Several studies, using transcranial magnetic stimulation (TMS) and transcranial direct current stimulation (tDCS), have typically targeted the prefrontal cortex (PFC), mainly the DLPFC, with the aim of reducing the excessive top-down cognitive control of AN patients and have shown positive effects on AN symptomatology and related behaviors [25, 26].

Across NIBS techniques, tDCS refers to the application of weak direct currents (0.5–2.0 mA) to a specific region of the brain, transmitted through electrodes attached to the scalp [27]. tDCS can be used to elicit an excitatory (anodal) or inhibitory (cathodal) effect, depending on the polarity of stimulation, and may induce long term potentiation (LTP) like plasticity [28, 29]. Compared with TMS, tDCS produces minor transient side-effects and is well tolerated by children and adults, with almost no discomfort and no limitation of movement [30–33], it is also less expensive, technically less demanding, and easy to transport and use in different environments, including the home by patients under medical supervision [34]. Considering the advantages of using tDCS, especially in the pediatric population, evidence of tDCS treatment efficacy may lead to important changes in the treatment of AN, with a substantial reduction in the times and costs of interventions.

However, while larger evidence exists from studies conducted with TMS [35–37], a few studies have applied tDCS for the treatment of AN with promising results. Khedr et al. [38] reported an improvement of depressive symptoms in adult patients with AN following 10 daily sessions of anodal tDCS over the left DLPFC (anode F3/cathode extracephalic—over the contralateral arm). Similarly, Strumila et al. [39] showed a reduction in eating and depressive symptoms following 20 sessions of tDCS over DLPFC (anode F3/cathode F4). In addition, data from an open-label study of our lab on adolescents with AN showed a positive effect of 18 sessions of left anodal/right cathodal tDCS to PFC, resulting in stable weight gain and improvement of psychopathological symptoms superior to a psychological control treatment [40]. However, the generalizability of these findings is low due to the lack of a sham control group and small sample size.

Despite some promising results, the effectiveness of tDCS for the treatment of AN symptoms is not always consistent. Baumann et al. [41] did not observe effect of active tDCS over the left DLPFC (anode F3/cathode over the right orbitofrontal region Fp2) on psychopathology and weight recovery in adult patients with AN, in a sham-controlled study. However, a reduction of the need to follow specific dietary rules and an improvement of body image evaluation were showed in the active tDCS group.

Although accumulating evidence suggests the presence of alterations in cortical excitation-inhibition balance in some mental disorders, such as AN [42–44], a definitive neurobiological under pinning of AN is lacking and few studies have directly investigated the brain mechanisms acting in AN in developmental age. In recent years, the integrative approach combining TMS with electroencephalography (EEG) has demonstrated to be a valuable tool to non-invasively probe brain circuits, allowing assessment of several cortical properties such as connectivity, plasticity, cortical excitability and inhibition [45–47]. Compared with other available neurophysiological methods, TMS-EEG responses are more sensitive to brain state and are influenced by brain maturation and ageing [48, 49]. As such, TMS-EEG can be applied in both basic science and clinical research [50]. Applied to various clinical populations, this technique may offer the opportunity to identify pathological biomarkers in brain dynamics which may supply new early tool of diagnosis and the identification of innovative therapeutic targets [51], as well as biomarkers to monitor treatment effects [52].

In addition, specific physiological biomarkers also need to be further investigated in order to assess possible effects of tDCS treatment on the vulnerability to stressors. Indeed, alterations in the functioning of the hypothalamic–pituitary–adrenal (HPA) axis, the main component of the endogenous stress response system, have been consistently reported in patients with AN [53] and appear to normalize in patients with weight recovery [54].

Overall, to explore the neurobiological mechanisms acting in AN, the use of brain-based approaches has been promoted [25, 26, 55] and large-scale, high-reproducibility clinical trials investigating the neurophysiological features of AN and the specific brain changes induced by NIBS are urgently needed.

### Objectives

We hypothesized that excitatory tDCS over the left PFC and inhibitory tDCS over the right PFC (anode left/cathode right) may aid in altering/resetting inter-hemispheric balance in children and adolescents with AN, reducing their control over eating behaviors and improving the AN psychopathology, assessed as changes in Eating Disorder Risk composite score (EDRC) of the Eating Disorder Inventory (EDI-3) as primary outcome. The study also aims to investigate some neurophysiological mechanisms that characterize AN, as well as the association between efficacy and neurophysiological effect of tDCS treatment and physiological response to treatment, as secondary outcome. We will employ TMS-EEG to directly explore inter-hemispheric balance in the DLPFC activity of children and adolescent with AN. Moreover, paired pulse TMS (pp-TMS) and repetitive TMS (rTMS) protocols will be used to investigate the functional mechanisms within the PFC of youth patients with AN. We hypothesized that at baseline there are differences between the right and left hemisphere in some electrophysiological measures such as inhibitory/excitatory motor circuits, sensory-motor integration, cortical plasticity, cortical oscillations, reactivity, and functional connectivity, and that such differences will be reduced after tDCS treatment. In addition, an increase in cortical plasticity after tDCS treatment is expected. Finally, we will assess if potential changes of specific biomarkers, such as those related to the endogenous stress response system functioning, nutritional status and body mass index (BMI), will occur after tDCS treatment and correlate with clinical improvement. Specifically, we hypothesized a reduction of endogenous stress response and an improvement of nutritional status and BMI after tDCS treatment.

In particular, this project has three different specific aims:

- *Specific Aim 1 “clinical efficacy”*: To evaluate the clinical efficacy of anodal/right cathodal tDCS to the PFC, coupled with a treatment-as-usual (TAU), in children and adolescents with AN in terms of: (1) changes in psychopathological measures, specifically changes in EDI-3 ED-specific (EDRC score) as primary outcome and changes in other psychopathological measures as secondary outcome; (2) changes in physiological measures, such as endogenous stress response system functioning, nutritional status, and BMI, as secondary outcome; (3) long-lasting effects until one year follow-up.
- *Specific Aim 2 “neurophysiological characterization at baseline”*: To characterize at baseline AN patients in terms of (1) intra-cortical inhibitory/excitatory motor circuits, and sensory-motor integration using pp-TMS; (2) cortical plasticity using rTMS; (3) cortical oscillations, reactivity, functional connectivity using TMS-EEG co-registration and to assess interhemispheric dynamics in terms of balance and inhibition.
- *Specific Aim 3 “association between efficacy and neurophysiological effect of tDCS treatment”*: To determine the neurophysiological patterns associated to behavioral changes induced by tDCS treatment, we will evaluate changes on plasticity and connectivity of the prefronto-motor networks, assessed by TMS-EEG, pp-TMS and rTMS, and their association to improvement.

### Trial design

The present randomized, double blind, placebo-controlled trial aims to evaluate the efficacy of a tDCS treatment in improving the clinical outcome of children and adolescents with AN, assessed as changes in Eating Disorder Risk composite score (EDRC) of the Eating Disorder Inventory (EDI-3).

## Materials and methods

### Study setting and participants

Eighty youth with AN will be recruited at the Anorexia and Eating Disorder simplex Unit, Child and Adolescent Neuropsychiatry Complex Unit, of the Bambino Gesù Children’s Hospital in Rome, Italy. Participants will be enrolled during the daily clinical activities of the Unit by a team of psychologists, neuropsychiatrists, and psychiatrists highly trained. Principal investigator will full inform participants and their parents about the procedures and purpose of the experiment, prior to obtain their written consent. Participation will be solely voluntary.

### Eligibility criteria

Inclusion criteria are the followings: 1. diagnosis of AN according to the Diagnostic and Statistical Manual of Mental Disorders, Fifth Edition—DSM-5 (American Psychiatric Association & American Psychiatric Association, 2013), confirmed by experienced developmental psychiatrists and psychologists through extensive clinical examination; 2. a condition of under-weight (BMI < 18.5 kg/m^2^); 3. intelligence quotient (IQ) higher or equal to 85 (IQ ≥ 85); 4. age ranging from 10 to 18 years included; 5. ability to give informed consent under parents' surveillance and guidance.

Exclusion criteria include: 1. a personal history of neurological/medical/genetic diseases; 2. a personal history of epilepsy; 3. suicide risk; 4. receiving CNS-active drug, other counseling or psychological therapies during the treatment.

### Interventions

All participants will undergo a tDCS treatment (active or sham) plus a TAU for six weeks, delivered for three times a week.

#### tDCS treatment

Participants in the active tDCS group will receive active stimulation to DLPFC via two saline-soaked, 25 cm^2^ sponges placed over F3 (anode) and F4 (cathode), according to the International 10–20 system. The current will be delivered via BrainStim stimulator (E.M.S. s.r.l.; Bologna, Italy) and will slowly increase during the first 30 s (ramp-up) to 1 mA while will decrease slowly to 0 mA during the last 30 s (ramp-down). Between the ramp-up and the ramp-down, a constant direct current (1 mA) will be delivered for 20 min, as in previous pediatrics tDCS studies [56–59]. To reduce the likelihood of irritation related to electrical stimulation, a low dose of gel cream (1/8 of an inch) will be applied on the sponges’ surface. Before tDCS application, the electrodes impedance will be checked to guarantee that it will be below 10 kΩ.

To control for any placebo effects, participants in the sham tDCS group will undergo the same procedures during the 20 min of the session, but the current will be applied for 30 s and will be ramped down without the participant’s awareness (0 mA).

To minimize any risk associated with tDCS, an experienced investigator will administer and supervise all tDCS sessions and ask the participant to report any discomfort. Stimulation stops if sensation on the scalp is uncomfortable or a headache occurs.

#### TAU

After each session of tDCS treatment, the participants will undergo TAU, including: 1. meetings for the nutritional and psychiatric monitoring for patients (once a week); 2. psychological support for patients by group sessions (twice a week, 60 min duration); 3. psychoeducation therapy for parents in group sessions (twice a week, 60 min duration).

Each session will be provided by highly trained professionals in ED (psychotherapists, psychiatrists and nutritionist), who will be blinded about the experimental conditions.

### Measures

To verify the efficacy of each condition (active or sham), a full assessment of psychological, neurophysiological and physiological measures will be carried at T0 and T1. Instead, follow-ups evaluations (T2, T3, T4, T5) will include only psychological and physiological assessment.

#### Psychological measures

Psychological measures about intellectual level [60] and socio-demographical status (parent-report questionnaire) will be collected at T0, while psychopathological measures will be collected at T0, T1 and at all follow-ups.

The psychopathological assessment of AN symptomatology will include: EDI-3 [61], which comprises 91-item that give a measure of basic ED characteristics through six composite scores [EDRC, Ineffectiveness (IC), Interpersonal Problems (IPC), Affective Problems (APC), Overcontrol (OC), and General Psychological Maladjustment (GPMC)] and nine general psychological scales [Low Self-Esteem; Personal Alienation; Interpersonal Insecurity; Interpersonal Alienation; Interoceptive Deficits; Emotional Dysregulation; Perfectionism; Asceticism; Maturity Fears]; Eating Attitudes Test (EAT-26) [62], which comprises 26-item that measures anorexia nervosa symptoms and produces a total score; and Body Uneasiness Test (BUT) [63], which contains 34-items to measures body image concerns and produces a global severity index (GSI). All questionnaires will be completed by the participants themselves.

To investigate participants’ behavioral and emotional symptoms, the Child Behavior Checklist for Ages 6–18 (CBCL 6–18) and the Youth Self-Report for Ages 11–18 (YSR 11–18) [64] will be administered, which are parental reports and self-reports questionnaires respectively. The CBCL 6–18 and YSR 11–18 questionnaires include a 113-item and 112-item scale, respectively, that produces several subscales, including syndrome scales (Withdrawn, Somatic Complaints, Anxious/Depressed, Social Problems, Thought Problems, Attention Problems, Delinquent Behavior and Aggressive Behavior, a Total Problem Score) and two broadband scores, Internalizing Problems and Externalizing Problems.

Anxiety and depressive symptoms will be evaluated through the following self-reports scales: Multidimensional Anxiety Scale for Children—second edition (MASC-2) [65] and the Children’s depression inventory—second edition (CDI-2) [66]. The MASC-2 is a 50-item self-report, which measure: Separation/Fears, Generalized Anxiety Index, Obsessions/Compulsions, Harm Avoidance, Social Anxiety (Humiliation/Rejection and Performance Fears) and Physical Symptoms (Panic and Tense/Restless). The CDI-2 is a 28-item self-report inventory that provides a Total Score and scores on two scales: Emotional Problems and Functional Problems. In addition, it provides scores for four further sub-scales, called Negative Mood/Physical Symptoms, Negative Self-Esteem, Ineffectiveness and Interpersonal Problems.

#### Neurophysiological measures

The neurophysiological measures will be collected at T0 and T1 in two separate sessions for each hemisphere respectively.

##### TMS protocols for the assessment of cortical excitability, sensory-motor integration and plasticity

Each TMS session consists of a pp-TMS and rTMS protocols. Specifically, pp-TMS protocols included short intracortical inhibition and facilitation (SICI/ICF) and short-latency afferent inhibition (SAI) with a conditioning-test design (magnetic or electric). Moreover, LTP mechanisms will be assessed in both hemispheres by rTMS, with the intermittent theta burst stimulation (iTBS) protocol. This measure allows to assess cortical excitability for each hemisphere, evaluated in terms of different motor-evoked potentials (MEPs) amplitude recorded at different time-points after rTMS perturbations. For each time point, these measures will be collected in two separate sessions, one day apart, for each hemisphere in order to avoid possible interhemispheric plasticity effects. Furthermore, iTBS protocol will be run at the end of the experimental session in order to avoid the interference of plasticity effect on other measures.

##### TMS-EEG protocol for the assessment of cortical oscillations, reactivity and functional connectivity

EEG was performed using a TMS-compatible EEG equipment (BrainAmp 32MRplus, 20 BrainProducts GmbH, Munich, Germany). In each TMS-EEG session, 80 TMS single pulses will be applied over the primary motor cortex (M1) and DLPFC of both hemispheres (320 pulses in total) at a random interstimulus interval (ISI) of 2 to 4 s, in counterbalanced order. TMS intensity will be set at 90% of the resting motor threshold (RMT) defined as the lowest intensity producing MEPs with a peak-to-peak amplitude > 50 µV in five out of 10 trials in the relaxed first dorsal interosseous muscle (FDI) of the hand [67]. TMS-evoked potentials (TEPs) will be evaluated in the spatiotemporal–domain to assess the cortical connectivity and reactivity measuring the waveform, latency, amplitude and cortical distribution of TEPs components. Moreover, the cortical oscillatory pattern will be explored with a time/frequency–domain analysis to evaluate the synchronization and desynchronization in theta, alpha and beta frequencies over motor and premotor areas.

In addition, quantitative EEG (qEEG) during resting state will be performed in an eyes-closed state at the beginning of the experimental session.

#### Physiological measures

Endogenous stress response system functioning and nutritional status will be collected at T0 and T1, as well as T2 and T4, while BMI will be assessed at each evaluation.

To investigate the endogenous stress response system functioning, the HPA axis functioning will be evaluated through the measurement of the Cortisol awakening response (CAR). To this purpose, participants will be instructed to collect saliva samples at home in two consecutive working days. They will be invited to collect saliva samples at awakening (in bed) and 15-, 30- and 60-min following awakening, by using the sampling device “Salivette” (Sarstedt; Rommelsdorft, Germany). Saliva cortisol concentrations will be measured by an enzyme immunoassay method, using a commercially available ELISA kit (Biochem Immunosystem, Milan, Italy). As measures of the CAR, the cortisol area under the curve relative to the ground (AUCg) and the AUC with respect to the increase (AUCi) will be also calculated.

The nutritional state will be evaluated by blood tests: Electrolytes, full blood count, Hematocrit, MCV, folate, B12, TIBC, albumin, transferrin, ESR, protein electrophoresis, serum total cholesterol, high density lipoproteins, triglycerides, glucose profile, glycaemia, insulin, AST, ALT, GGT, FT4, TSH, sexual hormones, cortisol, immunologic evaluation, copper.

Finally, BMI will be calculated as the person’s weight in kilograms divided by the square of height in meters.

#### Safety and tolerability questionnaire

Symptoms and side effects will be assessed using standard questionnaires [68] completed by participants after each tDCS session and at each follow-up. The questionnaire lists adverse effects, such as headache, neck pain, scalp pain, tingling, itching, burning sensation, skin redness, sleepiness, trouble concentrating, and acute mood change. Participants will quantify the intensity of the symptoms or side effects that are related to tDCS as follows: (1) absent; (2) mild; (3) moderate; and (4) severe.

To screen potential subjects for risk of adverse events during TMS, all participants completed non-invasive brain stimulation screening [69] that consists of fourteen yes or no questions, based on accepted safety considerations for TMS.

### Outcomes measures

#### Primary outcome measure

We believe that active tDCS, coupled with TAU, will enhance outcome of traditional treatment and will have a substantial impact on symptoms recovery of AN.

The primary end-point of the study is significant improvement on AN psychopathology at T1, assessed as changes in EDI-3 ED-specific (EDRC score).

#### Secondary outcome measures

Secondary psychological outcome measures will be collected at T1, T2, T3, T4 and T5, physiological outcome will be collected at T1, T2 and T4 and only BMI also at T3 and T5, instead neurophysiological outcome measures will be collected only at T1.

Improvement on the other EDI-3 composite scores, EAT-26 total score, BUT-GSI, and CBCL 6–18, YSR 11–18, MASC-2 and CDI-2 subscale’s t-scores, will be considered as secondary psychological end-points.

Possible reduction of left and right hemisphere baseline differences in TEPs, in SICI/ICF and SAI protocols, and increase MEP amplitude in iTBS protocol will be considered as neurophysiological secondary end-points.

Finally, reduction in the CAR, CAR AUCg and AUCi and improvement in nutritional state and BMI, which have normative data for age and sex, will be adopted as secondary physiological end-points.

See Table 1 for details on the outcome measures.

**Table 1.** Outcome measures.

Primary outcome measure	Secondary outcome measures	
EDRC score (EDI-3)	Psychopatological	IC, IPC, APC, OC, GPMC scores (EDI-3) EAT-26 total score BUT-GSI CBCL 6–18 subscale’s t-scores YSR 11–18 subscale’s t-scores MASC-2 subscale’s t-scores CDI-2 subscale’s t-scores
	Neuropsysiological	TEPs, SICI/ICF and SAI protocols MEP amplitude in iTBS protocol
	Physiological	CAR, CAR AUCg and AUCi Nutritional state and BMI

*APC* Affective problems, *BMI* Body Mass Index, *BUT* Body Uneasiness Test, *CBCL 6–18* Child behavior checklist for ages 6–18, *CDI-2* Children’s depression inventory—second edition, *AUCg* Cortisol area under the curve relative to the ground, *AUCi* Cortisol area under the curve with respect to the increase, *CAR* Cortisol awakening response, *EAT-26* Eating Attitudes Test, *EDI-3* Eating Disorder Inventory, *EDRC* Eating Disorder Risk, *GPMC* General Psychological Maladjustment, *GSI* global severity index, *IC* Ineffectiveness, *IPC* Interpersonal problems, *MEPs* Motor-Evoked Potentials, *MASC-2* Multidimensional Anxiety Scale For Children—second edition, *OC* Overcontrol, *SICI/ICF* Short intracortical inhibition and facilitation, *SAI* Short-latency afferent inhibition, *iTBS* Theta burst stimulation, *TEPs* TMS-evoked potentials, *YSR 11–18* Youth Self-Report for Ages 11–18.

### Participant timeline

Baseline assessment (at time 0-T0) will be completed before the interventions are administered. Participants will undertake tDCS treatments (active or sham) plus a TAU for six weeks delivered for three times a week (18 session). Participants will be evaluated at the end of the treatment (at time 1-T1) and one-month later (at time 2-T2), three months later (at time 3-T3), six months later (at time 4-T4) and one year later (at time 5-T5). In the last follow-up, participants will be asked to indicate whether they believe they were receiving active or sham tDCS.

**Fig. 1:**
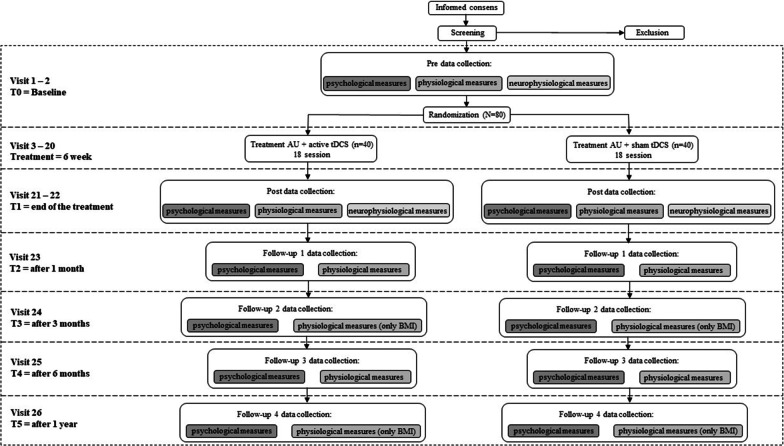
Study procedure and assessments. *AU* as usual, *tDCS* transcranial direct current stimulation, *BMI* Body Mass Index

See Fig. 1 for details on the specific assessment of each visit.

### Sample size

The sample size was calculated by a priori analysis in G * Power, version 3.1.9.7 (The G*Power Team, Düsseldorf, Germany).

Assuming a correlation of 0.80 in the primary outcome measure (EDRC score of EDI-3 questionnaire) with an estimated f = 0.08, α value = 0.05 (i.e., probability of false positives of 5%), and β = 0.80 (i.e., at least 80% power), the sample size that was required for repeated-measures analysis of variance (ANOVA) with 2 groups (active tDCS vs sham tDCS) and 6 measurements (T0 vs. T1 vs. T2 vs. T3 vs. T4 vs. T5) was 68 (i.e., 34 per group). Considering a 15% dropout rate in the follow-ups, we will plan to recruit a total of 80 participants (i.e., 40 per group).

### Allocation

Before the stratified randomization, screening for clinical eligibility will be completed by a trained experimenter.

Stratified randomization will be performed via the minimal sufficient balancing method to prevent disparities at baseline. Namely, age, BMI and AN severity assessed by the EDI-3, will be taken in to account. Participants will be randomly assigned to two treatment groups: 1. TAU plus active tDCS (experimental treatment); 2. TAU plus sham tDCS (control treatment).

The stratified randomization will be carried out by an independent researcher.

### Blinding

The investigators, the highly trained ED professionals involved in the TAU and participants, as well as their parents, will be blinded to the group allocation. In addition, data for each measure will be collected from blinded experimenters and will be insert in electronic and protected data files.

Randomization information will be maintained until data collection is completed by an independent researcher, who will have an emergency code break envelope to open in case of serious adverse event that requires knowledge of the interventions to manage the participant’s condition.

### Safety considerations

The risks associated with participation in the study are low. One possible critical aspect is related to the use of NIBS itself. Standard NIBS paradigms are considered safe and well-tolerated in developmental age [70] including those with underlying neurological conditions or mental disorders [71]. Safety guidelines for the application of TMS and tDCS in pediatric patients report no serious adverse events, but only mild and transient [34, 70, 72].

Previous studies conducted by our laboratory [40, 56–59, 73] have also demonstrated the safety and tolerability of tDCS and its usefulness in improving cognitive and psychopathological measures in children and adolescents with developmental and psychopathological disorders.

Adverse effects will be registered during the total time of the study, using a standard questionnaire [68, 69]. The experimenter will follow participants for adverse effects even after the end of the study.

### Study monitoring and data management

The principal investigator (or the ethics committee) will identify a study monitor assigned to follow this study in accordance with this Clinical Trial Protocol [European guidelines for Good Clinical Practice (CPMP/ICH/135/1995) and Decree-Law Italian Minister of Health, 15 July 1997]. The Investigator agrees to provide reliable data and all information requested by the Protocol (with the help of the Case Report Form (CRF), or other appropriate instruments) in an accurate and legible manner according to the instructions provided and to ensure direct access to source documents to the ethics committee representatives. If any particular circuits have to be defined (e.g., e-CRF, Fax), particular attention should be paid to the confidentiality of the patient’s data to be transferred.

The principal investigator may appoint such other individuals as he/she may deem appropriate as Sub-Investigators to assist in the conduct of the Clinical Trial in accordance with the Clinical Trial Protocol. All Sub-Investigators shall be timely appointed and listed. The Sub-Investigators will be supervised by and under the responsibility of the Investigator. The Investigator will provide them with a Clinical Trial Protocol and all necessary information.

The participants’ personal data will be anonymous and coded. The hard files will be placed in a closed drawer. The database will be protected by password. The investigators will allow the monitoring of the data at an appropriate frequency. The original documents will be available at any time to be verified by the clinical monitor and regulatory authority.

### Statistical methods

The Shapiro–Wilk test will be used to test the normality of the data and Levene’s test for the homogeneity of variances. When data will be normally distributed and the assumption of homogeneity will be not violated, parametric analyses will be computed. When one assumption will be not meet, non-parametric tests will be conducted or a log-transformation of distribution will be applied, if appropriate.

The groups will be compared on demographic and categorical variables using Chi-Square analyses. The primary outcome (EDRC score of EDI-3) will be included in a General Linear Model and will be analyzed by means of repeated measures ANOVA with Group (active tDCS + TAU vs sham tDCS + TAU) as between-factor and Follow-ups (T0-T1 vs T0-T2 vs T0-T3 vs T0-T4 vs T0-T5) as within-factor. The same analyses will be applied to the secondary psychological outcomes (EAT-26 total score, BUT-GSI, and CBCL 6–18, YSR 11–18, MASC-2 and CDI-2 subscale’s t-scores). Post hoc comparisons will be run by means of Bonferroni test.

For secondary neurophysiological outcomes, the differences between the left and right hemisphere at baseline and between the recordings T0 and T1 will be evaluated on both TEPs component and frequency through a non-parametric cluster based permutation tests to correct for multiple comparisons as developed in the ft_timelockstatistics\ ft_freqstatistics functions in Fieldtrip [74]. The EEG signal will be analyzed for defining a resting state pattern with frequency inspection, using a Fourier analysis. For TMS data, prior to undergoing ANOVA, the normal distribution of data will be assessed by means of the Shapiro-Wilks test. The level of significance was set at 0.05. For the SICI, SAI, and iTBS protocols, we will perform three ANOVAs on the normalized values calculated as the percentage of the mean peak-to-peak amplitude size of the unconditioned TS with GROUP (active vs sham) as the between-subject factor and ISI (for SICI and SAI protocols) or TIME (for iTBS protocol) as within-subject factors. When a significant main effect will be reached, Bonferroni post hoc comparisons will be performed to characterize the specific effect.

For secondary physiological outcomes, differences in saliva CAR, CAR AUCg and AUCi between groups (active tDCS + TAU vs sham tDCS + TAU) will be evaluated by repeated measures ANOVAs with time (T0 vs T1 vs T2 vs T4) as within-subject factors; and differences in BMI between groups (active tDCS + TAU vs sham tDCS + TAU) will be evaluated by repeated measures ANOVAs with time (T0 vs T1 vs T2 vs T3 vs T4 vs T5) as within-subject factors.

### Ethics

Ethical approval for the study was granted by the local research ethics committee (process number 763_OPBG_2014) and was registered at ClinicalTrials.gov (ID: NCT05674266). This study will be performed in accordance with the Declaration of Helsinki. The present study protocol adheres to the SPIRIT guidelines (Standard Protocol Items: Recommendations for Interventional Trials) and was prepared using the SPIRIT 2013 Checklist.

## Discussion

AN has a high impact on both the individual, family and society [9]. Given the poor outcome of available treatments for AN, novel approaches have been called for.

We have described the rationale and design of a trial that aims to determine the effect of treatment based on NIBS in improving the clinical outcome of traditional treatment in children and adolescents with AN. This study will constitute one of the first attempt to prove the clinical efficacy of multiple tDCS sessions in the pediatric population with AN, evidencing neurophysiological and physiological correlations. Furthermore, the present project also aims to explore brain functional characteristics of youth with AN including plasticity, connectivity and interhemispheric balance.

We chose to implement a multi-sessions protocol because it has been documented that higher number of tDCS sessions correlates with greater behavioral changes [75]. The selection of tDCS parameters was based on our previous study, showing beneficial and safe effects of tDCS treatment in adolescent with AN [40]. The decision to apply 1 mA was made considering the guidelines for children that recommend applying at least half of that for adults [76]. Indeed, in adult 2 mA is well tolerated without adverse effects [25, 26], but in pediatric population the less cerebrospinal fluid and the smaller head size should be considered [76–78].

A point of strength of this study relies on the use of neurorehabilitative approach that has the potential to target brain abnormalities through plasticity mechanisms, essential in development age. Indeed, in the developmental age brain plasticity is characterized by a maximal state of synaptic pruning and axonal myelination [79, 80], so responsiveness to interventions in this period is increased [81, 82].

An additional aspect is the multilevel assessment to detect the direct effects of tDCS treatment involving psychological, neurophysiological, and physiological levels. To evaluate the impact of tDCS treatment to improve psychopathological symptoms of participants, we have chosen the variance on EDI-3 ED-specific (EDRC score) as primary end-point. To date, the literature on clinical trials for AN considers psychopathological improvement, along with improvement in BMI, as the optimal outcome for AN correction [83–85]. Indeed, evidence from NIBS indicated that the improvement on self-reported symptoms does not always translate into weight gain [86]. Furthermore, often an improvement on weight without the same improve on cognitive and behaviors symptoms represented a negatively experienced in people with AN affecting their self-esteem [87]. Moreover, considering that brain mechanisms acting in AN are poorly understood, recording of TMS, EEG, and TMS-EEG data before and after the tDCS treatment will be used as a proxy of brain plasticity and as a reliable neurophysiological marker for treatment responders. Lastly, identifying possible biomarkers of the response to treatment, such as the HPA axis functioning, would represent an important step in the progress towards precision and personalized medicine in AN [88].

We believe that this clinical trial will provide the scientific basis to accelerate the validation of brain-based treatments for AN in development and could lead to important changes in the treatment of AN.

## Data Availability

Not applicable.

## Abbreviations

APC: Affective problems
ANOVA: Analysis of variance
AN: Anorexia Nervosa
BMI: Body mass index
BUT: Body Uneasiness Test
CRF: Case Report Form
CBCL 6–18: Child Behavior Checklist for Ages 6–18
CDI-2: Children’s Depression Inventory—second edition
AUCg: Cortisol area under the curve relative to the ground
AUCi: Cortisol area under the curve with respect to the increase
CAR: Cortisol awakening response
DLPFC: Dorsolateral prefrontal cortex
EAT-26: Eating attitudes test
ED: Eating disorder
EDI-3: Eating Disorder Inventory
EDRC: Eating Disorder Risk
EEG: Electroencephalography
GPMC: General Psychological Maladjustment
GSI: Global severity index
HPA: Hypothalamus pituitary adrenal
IC: Ineffectiveness
IQ: Intelligence quotient
IPC: Interpersonal problems
ISI: Interstimulus interval
LTP: Long term potentiation
MEPs: Motor-evoked potentials
MASC-2: Multidimensional Anxiety Scale for Children—second edition
NIBS: Non-invasive brain stimulation techniques
OC: Overcontrol
ppTMS: Paired pulse TMS
PFC: Prefrontal cortex
qEEG: Quantitative EEG
rTMS: Repetitive TMS
RMT: Resting motor threshold
SICI/ICF: Short intracortical inhibition and facilitation
SAI: Short-latency afferent inhibition
iTBS: Theta burst stimulation
TEPs: TMS-evoked potentials
tDCS: Transcranial direct current stimulation
TMS: Transcranial magnetic stimulation
TASS: Transcranial magnetic stimulation adult safety screen
TAU: Treatment-as-usual
YSR 11–18: Youth Self-Report for Ages 11–18
